# Phytochemicals from Ajwa dates pulp extract induce apoptosis in human triple-negative breast cancer by inhibiting AKT/mTOR pathway and modulating Bcl-2 family proteins

**DOI:** 10.1038/s41598-021-89420-z

**Published:** 2021-05-14

**Authors:** Mohsin Ali Khan, Sahabjada Siddiqui, Imran Ahmad, Romila Singh, Durga Prasad Mishra, Anand Narain Srivastava, Rumana Ahmad

**Affiliations:** 1 Chancellor, Era’s Lucknow Medical College and Hospital, Era University, Lucknow, 226003 India; 2 Department of Biotechnology, Era’s Lucknow Medical College and Hospital, Era University, Lucknow, 226003 India; 3 Environmental Toxicology Group, CSIR-Indian Institute of Toxicology Research, Lucknow, 226001 India; 4 Cell Death Research Laboratory, LSS-106, Endocrinology Division, CSIR-Central Drug Research Institute, Lucknow, 226031 India; 5 Department of Pathology, Era’s Lucknow Medical College and Hospital, Era University, Lucknow, 226003 India; 6 Department of Biochemistry, Era’s Lucknow Medical College and Hospital, Era University, Lucknow, 226003 India

**Keywords:** Cancer, Drug discovery, Molecular biology, Oncology

## Abstract

Ajwa dates (*Phoenix dactylifera* L.) have been described in traditional and alternative medicine to provide several health benefits, but their mechanism of apoptosis induction against human triple-negative breast cancer MDA-MB-231 cells remains to be investigated. In this study, we analyzed the phytoconstituents in ethanolic Ajwa Dates Pulp Extract (ADPE) by liquid chromatography-mass spectrometry (LC–MS) and investigated anticancer effects against MDA-MB-231 cells. LC–MS analysis revealed that ADPE contained phytocomponents belonging to classes such as carbohydrates, phenolics, flavonoids and terpenoids. MTT assay demonstrated statistically significant dose- and time-dependent inhibition of MDA-MB-231 cells with IC_50_ values of 17.45 and 16.67 mg/mL at 24 and 48 h, respectively. Hoechst 33342 dye and DNA fragmentation data showed apoptotic cell death while AO/PI and Annexin V-FITC data revealed cells in late apoptosis at higher doses of ADPE. More importantly, ADPE prompted reactive oxygen species (ROS) induced alterations in mitochondrial membrane potential (MMP) in ADPE treated MDA-MB-231 cells. Cell cycle analysis demonstrated that ADPE induced cell arrest in S and G2/M checkpoints. ADPE upregulated the p53, Bax and cleaved caspase-3, thereby leading to the downregulation of Bcl-2 and AKT/mTOR pathway. ADPE did not show any significant toxicity on normal human peripheral blood mononuclear cells which suggests its safe application to biological systems under study. Thus, ADPE has the potential to be used as an adjunct to the mainline of treatment against breast cancer.

## Introduction

Breast cancer is an abnormal growth of cells lining the breast lobules or ducts. These cells grow uncontrollably and have the potential to spread to other parts of the body. Breast cancer impacts 2.1 million women each year and is also responsible for  the greatest number of cancer-related deaths among women. Though the prevalence of breast cancer in Asia is less than that in Western countries, but in modern times, the relative contribution to the global burden of breast cancer is rising rapidly in Asia^1^. In 2019, there was an estimation of 268,600 new cases of invasive breast cancer among females to be reported in the US and 41,760 estimated death due to breast cancer^2^. Breast cancer is the most common cancer of females in India, and  accounts for 25–32% of female cancers in India. One woman is diagnosed with breast cancer every 4 min and one woman dies of breast cancer every 13 min in India^3^. Breast cancer projection is expected to go as high as 17,97,900 in 2020^4^.

At present, various treatments including surgical resection, chemotherapy, radiation therapy, hormonal therapy and synthetic lethality have been developed. However, surgical resection at the metastasis stage often limits its effectiveness and chemotherapy and radiotherapy can have a negative effect on normal cells^5^. Therefore, treatment with insignificant adverse effects, specifically killing cancer cells with minimal toxicity to normal cells may present as a practical approach for improving breast cancer therapy. Targeted molecular therapies that block tumor cell proliferation and are less toxic to normal cells, may contribute to improving survival and quality of life in breast cancer patients^6,7^.

Phytonutrients like carotenoids, isothiocynates, phenolic compounds, flavanoids, organo-sulfides, isoflavones and indoles found in plant-based foods such as fruits, vegetables, beans and grains may help to prevent and fight breast cancer. Foods that are rich in fiber, such as whole grains, beans, and legumes may also help in fighting breast cancer. Ajwa dates being a rich source of antioxidants, flavonoids, as well as fiber can serve as a good anticancer agent against breast cancer. Ajwa dates (*Phoenix dactylifera* L.), belonging to family Arecaceae contain polyphenolics and other bioactive compounds which are used in traditional remedies for potential prevention of cell damage and cancer therapeutics^8^. Date fruits have been consumed in Arab and its neighboring areas since time immemorial as part of the essential diet. Recent research has shown the anticancer potential of Ajwa dates extract in combination with 5-fluorouracil against human breast adenocarcinoma cell line MCF-7^9^ and apoptosis-inducing effect and cell cycle arrest in prostate cancer cells PC3^10^. The ethnopharmacological significance of date palm is well- described in terms of its antioxidant, anti-inflammatory and antitumoral activities against breast carcinoma cells^11^. Other parts of the date palm have also been found to exhibit potent anticancer activity *viz.* seed extract against azoxymethane-induced colon carcinoma in rats, root hair extract in combination with silver nanoparticles^12,^ palm pollen extract in combination with silver and gold nanoparticles against breast adenocarcinoma cells^13^ and leaf extract against human malignant melanoma cell line IGR-39^14^. However, none of the studies have, so for, reported the mechanism of anticancer action of Ajwa dates pulp extract by modulation of Bcl-2 family proteins and downregulation of AKT/mTOR signaling pathway in breast carcinoma MDA-MB-231 cells.

In the present study, MDA-MB-231 cell line was treated with various concentrations of ADPE and was analyzed for the underlying regulatory mechanism(s) of apoptotic cell death. To identify major phytoconstituents in ADPE, phytochemical characterization of ADPE was done using LC–MS. ADPE was also tested for its toxicity against normal human peripheral blood mononuclear cells (PBMCs).

## Materials and Methods

### Reagents and chemicals

Dulbecco’s Modified Eagle Medium Nutrient Mixture F-12 (DMEM/F-12) and antibiotic–antimycotic (penicillin G and streptomycin) solution were purchased from Gibco, Invitrogen, USA. Fetal bovine serum (FBS) was procured from Himedia, India. Hoechst 33342, propidium iodide (PI), Ribonuclease A (RNase A), 2,7-dichlorodihydrofluorescein diacetate (DCFH-DA), acridine orange (AO), ethidium bromide (EtBr) and rhodamine 123 (Rh 123) were purchased from Himedia, India. Annexin V-fluorescein isothiocyanate (FITC)/propidium iodide (PI) apoptosis detection kit was supplied by BioVision, USA. The antibodies used in western blotting against β-actin, p53, Bax, Bcl-2, cleaved caspase-3, AKT, p-AKT^473^, mTOR, p-mTOR^2448^ were purchased from Cell Signaling Technology (Danvers, MA). Other chemicals were obtained from Sigma-Aldrich, USA and were of analytical grade.

### Preparation of Ajwa dates extract

The present study was carried out at Cell and Tissue Culture Lab, Department of Biochemistry, Era's Lucknow Medical College and Hospital, Era University, Lucknow. Fresh Ajwa dates were procured from Al-Madina Al-Munawwarah, Kingdom of Saudi Arabia. To extract phytocomponents, 95% ethanol was used as a solubilizing agent that dissolves various semi-polar compounds such as sterols, flavonoids, phenolics, hydroquinones and alkaloids. Ethanol is a solvent having lesser polarity as compared to water making it an ideal solvent for extraction of both polar and non polar compounds. Briefly, pulp part of date fruit was manually separated, washed with double distilled water, sun-dried and coarsely powdered using pestle and mortar. The coarse powder contents were then extracted in 95% ethanol (1:3 ratio, weight by volume) at 25 °C for 3 days. The extracted solvents were pooled and filtered through Whatman No. 1 filter paper (125 mm). The filtrate obtained was concentrated in vacuum at 45 °C using Rotavapor (Buchi Rotavapor R-205, Switzerland). The obtained extract was further concentrated in a water bath until a semi-solid paste was obtained and stored in an air-tight container at room temperature until further use in experiments.

### Culture of human breast cancer cell line and PBMCs

Human triple negative breast cancer cell line MDA-MB-231 was procured from the cell repository center of the National Centre for Cell Sciences, Pune, India. Human PBMCs were separated from heparinized blood of a healthy volunteer who provided informed consent for participation in this study. The experimental protocol was approved by the Institutional Ethics Committee, Era’s Lucknow Medical College and Hospital, Era University, Lucknow, India. All of the experimental protocols were carried out in accordance with the guidelines of Institutional Ethics Committee, Era’s Lucknow Medical College and Hospital, Lucknow, Era University, India and Central Drugs Standard Control Organization, FDA Bhawan, New Delhi, India. MDA-MB-231 cells were maintained in DMEM/F-12 (1:1) media while PBMCs were cultured in RPMI-1640 media supplemented with 10% heat-inactivated FBS, 2 mM L-glutamine, 1% penicillin G and streptomycin solution and maintained at 37 °C with 5% CO_2_ in an incubator (Thermo Scientific, USA).

### Phytochemical characterization of ADPE by LC–MS

ADPE was subjected to phytochemical analysis by high-performance liquid chromatography (HPLC) coupled with mass spectrometry (MS) on a triple quadrupole tandem mass spectrometer (ACQ-TQD#QBB1152). LC was performed on a C18 reverse-phase column (150 × 2.1, 2.6 μm) and elution was done for 30 min, using mobile phase solvent  acetonitrile/water (5:95, v/v), acetonitrile, methanol and 5 mM ammonium acetate (95:5 H_2_O:Acetonitrile, pH 6.5). The mobile phase was kept at a flow ramp rate of 0.45 mL min^-1^ and the sample injection volume was 5 μL. Waters Acquity PDA detector type- UPLC eLambda 800 nm was used at wavelength range 210–800 nm and resolution 1.2 nm. The spectrometer was operated in positive and negative modes. The source temperature was 120 °C, the desolvation temperature was 350 °C and the cone voltage was set at 30 V. MS data was recorded in the mass range, m/z 200–2000 from 0–30 min under ionization mode of ES^+^ and ES^-^. All eluted peaks from HPLC were recorded at different retention times. The fractions were then characterized by mass spectrometry. This analysis was carried out at the Sophisticated Analytical Instrumentation Facility (SAIF), CSIR- Central Drug Research Institute (CDRI), Lucknow, India.

### MTT assay on breast cancer cells

The cell viability of ADPE against breast cancer cells was evaluated by MTT reduction assay following a previously published protocol^15^. MDA-MB-231 cells were seeded at a density of 1 × 10^4^ cells/well in a 96-well microtiter culture plate and incubated overnight. ADPE was diluted in culture media and treated in triplicate with varying concentrations (10, 12, 15, 18, 20, 22 and 25 mg/mL) of ADPE for 24 and 48 h. The absorbance values were read in an ELISA plate reader (BIORAD-PW41, USA) at 550 nm with a reference wavelength of 630 nm. The cellular morphological changes were observed under an inverted phase-contrast microscope (Nikon Eclipse TS100, Japan).

### Cytotoxicity against PBMCs

Human PBMCs were isolated from heparinized blood using Histopaque-1077 density gradient (Sigma Aldrich, USA) following a previous protocol with slight modification^16^. Briefly, fresh blood was diluted with equal volume of PBS (1X) and lightly poured over equal volume of Histopaque 1077 in 15 ml falcon tube followed by centrifugation at 1500 rpm for 15 min at 20 °C in a hanging rotor centrifuge. The middle buffy-coat containing mononuclear cells was collected in a fresh tube and washed twice with PBS. For toxicity assay, 1 × 10^5^ PBMCs/well were seeded into nutrient media in a 96-well culture plate. After 2 h, different concentrations of ADPE were added in each well in triplicate except the control wells and incubated for 24 h. Absorbance and percentage of cytotoxicity were determined as mentioned above using ELISA plate reader (BIORAD-PW41, USA).

### Nuclear condensation assay

Based on the cell viability assay, the apoptosis-inducing effect of ADPE was evaluated at three effective doses *viz.* 15, 18 and 20 mg/mL on MDA-MB-231 cells. DNA condensation was measured using Hoechst 33342 staining as per a previously published method^17^. To assess nuclear morphology, stained cells were captured under an inverted fluorescence phase-contrast microscope (Zeiss AxioVert 135, USA).

### Acridine orange-ethidium bromide (AO/EtBr) assay

The mechanism of cytotoxicity of ADPE on MDA-MB-231 cells was evaluated as reported previously^18^. MDA-MB-231 cells were seeded in a 24-well culture plate and treated at 15 and 25 mg/mL of ADPE for 48 h. The cells were stained with AO/EtBr (2 µg/mL each) fluorescent dyes for 10 min at 37 °C in a CO_2_ incubator. Subsequently, cells were washed twice with ice-cold phosphate buffer saline (PBS) and observed under an inverted fluorescence phase-contrast microscope (Zeiss AxioVert 135, US).

### DNA fragmentation assay

Genomic DNA was isolated from both treated and untreated cells as per the instruction manual of NucleoSpin Blood Kit (Macherey–Nagel, Germany). Briefly, MDA-MB-231 cells at a density 1 × 10^6^ were cultured in T-25 cm^2^ culture flasks overnight and cells were then treated with different concentrations of ADPE for 48 h. Treated cells were washed with PBS and resuspended in 200 µL PBS. Genomic DNA was isolated from cultured cells as per the protocol of the NucleoSpin Blood Kit. Electrophoresis of extracted DNA was performed on 1.5% agarose gel at 60 V for 120 min using 1X TBE buffer in a gel electrophoresis unit (Genei, India). DNA bands were observed under ultraviolet illumination gel-doc system (BIORAD, USA).

### Analysis of apoptosis by Annexin V-FITC double stain

Apoptotic cells were quantified with an Annexin V-FITC Apoptosis Kit (BioVision, USA) according to the manufacturer’s protocol using flow cytometry. Briefly, cells at 1 × 10^6^ cells/mL density were incubated with 15, 18 and 20 mg/mL concentrations of ADPE for 48 h. Cells were then harvested and re-suspended in the binding buffer and stained with 2 μLAnnexin V-FITC and 2 μL PI for 15 min at 25 °C in dark. The apoptotic index was immediately analyzed by flow cytometry (FACS Canto II flow cytometer, BD Biosciences, USA).

### Intracellular ROS measurement

Intracellular ROS levels were estimated using DCFH-DA dye by fluorescence microscopy imaging and flow cytometry techniques as reported previously^15^. The intracellular fluorescence intensity of cells was visualized by using an inverted fluorescence microscope. To quantify ROS intensity, both treated and untreated cells were harvested and washed with PBS and incubated in PBS containing 10 µM DCFH-DA dye at 37 °C for 20 min. The cells were then washed twice with PBS and subjected to flow cytometry analysis.

### Mitochondrial membrane potential (MMP, ΔΨ_m_) measurement

The alterations in MMP were assessed with the fluorescent probe Rh123 as per the previously published protocol^19^. The images of incubated cells were captured under a fluorescence microscope. For flow cytometry analysis, treated cells were incubated with Rh123 at a final concentration of 10 µM for 30 min in the dark. After washing with PBS twice, cells were resuspended in 500 µl PBS and analyzed using flow cytometry.

### Analysis of cellular DNA content

Cells were seeded at density 1 × 10^6^ cells/mL into 6-well plate and treated with ADPE (15, 18 and 20 mg/mL) for 48 h. Different phases of the cell cycle with cellular DNA contents were analyzed using flow cytometry as described previously^15^.

### Western blot analysis

Western blotting was carried out as per a previously standardized protocol^20^. Briefly, MDA-MB-231 cells at a density of 1 × 10^6^ in T-25 cm^2^ flask were treated with two effective doses *viz.* 15 and 20 mg/mL of ADPE. Based on the IC_50_ value (16.67 mg/mL) of ADPE after 48 h, these two effective doses, one below IC_50_ and the other above IC_50_, were selected to show their differential effects on selected apoptotic markers in MDA-MB-231 cells. The whole cell lysates were prepared by scrapping the cells in ice-cold RIPA lysis buffer supplemented with protease and phosphatase inhibitor cocktail (Thermo Fisher Scientific, USA). The lysates from each sample were centrifuged at 13,000×g for 20 min and the protein concentration in the supernatant was determined with a BCA protein assay kit (Thermo Fisher Scientific, USA) as per manual instruction. Equal amounts (30 μg) from each sample of protein lysate were subjected to SDS-PAGE on a 6% gel for mTOR/p-MTOR and a 12% gel for AKT/p-AKT, p53, Bax, Bcl-2, cleaved caspase-3. Mini Trans-Blot Module (BIORAD) was used to electrotransfer separated proteins to a PVDF membrane and thereafter the blot was blocked with blocking buffer containing 5% BSA in Tris Buffer Saline Tween 20 (TBST) solution, pH 7.4 under constant agitation for 1 h at 4 °C. Membranes were then incubated with primary antibodies overnight at 4 °C. Following washing with TBST thrice, membranes were incubated with horseradish peroxidase (HRP)- conjugated secondary antibodies for 1 h at RT with gentle shaking. After washing, bands were visualized by ECL Western Blotting Substrate Kit (Thermo Fischer Scientific, USA) according to the manufacturer’s instructions. The relative abundance of each band was quantified using Image J software (version 1.43, NIH, USA) and normalized to β-actin as a loading control.

### Statistical analysis

Cell viability data were expressed as the mean ± SEM from three independent experiments. Statistical evaluation was determined by one-way ANOVA followed by Dunnett’s Multiple Comparison Test using GraphPad Prism software (Version 5.01). A *p-*value of less than 0.05 was considered statistically significant.

## Results

### Identification of phytocomponents in ADPE using LC-MS

**Figure 1:**
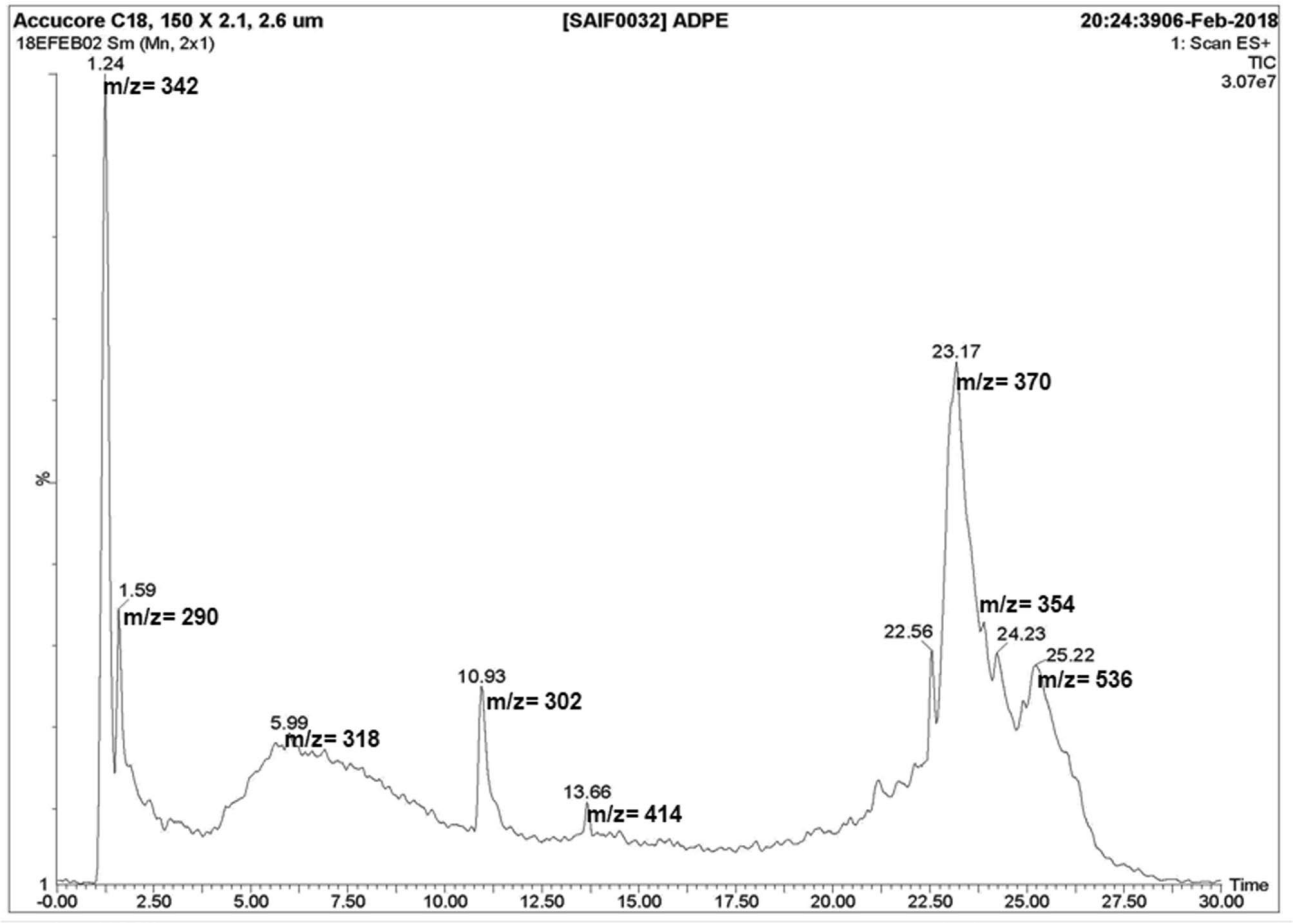
Total Ion Chromatogram (TIC) of 95% ethanolic ADPE obtained using LC–MS. A 5 mL sample was loaded on Silica C18 Reversed Phase column (150 × 2.1, 2.6 μm), with 0.45 mL min^−1^ flow rate using mobile phase solvents (**a**): acetonitrile/water (5:95, v/v), (**b**): acetonitrile, (**c**): methanol and (**d**): 5 mM ammonium acetate (95:5 H_2_O:Acetonitrile, pH 6.5). Mass spectrometry was performed by ESI and MS data was recorded in the mass range m/z 200–2000 from 0–30 min under ionization mode of ES^+^ and ES^−^. Y-axis is relative abundance and X-axis is retention time.

Most probable adducts were recognized to be [M + H]^+^, [M + Na]^+^, [M + K]^+^ and [M + K + 2H]^+^ which were used for analysis of MS data qualitatively. The names, MWs and retention time (R_t_) of compounds contained in ADPE were ascertained. LC–MS chromatogram of ADPE showed eight peaks indicative of presence of eight bioactive components (Fig. 1). On comparison of the mass spectra of the constituents, eight phytocomponents were characterized and identified as shown in Table 1. The compounds having the highest peak were identified as maltose, catechin, myricetin, quercetin, β-sitosterol, digalacturonic acid, chlorogenic acid and β-carotene.

**Table 1:** Name of phytocomponents, molecular formula, MW and structure of different phytoconstituents identified from ethanolic ADPE using LC-ESI/MS.

S. No	Name of phytocomponent	Structure	Formula	MW	Retention time (R_t_) in min	Chemical class
1.	Maltose		C_12_H_22_O_11_	342	1.24	Disaccharide
2.	Catechin		C_15_H_14_O_6_	290	1.59	Flavonoid
3.	Myricetin		C_15_H_10_O_8_	318	5.99	Flavonoid
4.	Quercetin		C_15_H_10_O_7_	302	10.93	Flavonoid
5.	β-sitosterol		C_29_H_50_O	414	13.66	Phytosterol
6.	Digalacturonic acid		C_12_H_18_O_13_	370	23.17	Disaccharide derivative
7.	Chlorogenic acid		C_16_H_18_O_9_	354	24.23	Phenolic
8.	β-Carotene		C_40_H_56_	536	25.22	Carotenoid

### Effect of ADPE on cell viability of MDA-MB-231 cells and human PBMCs

**Figure 2:**
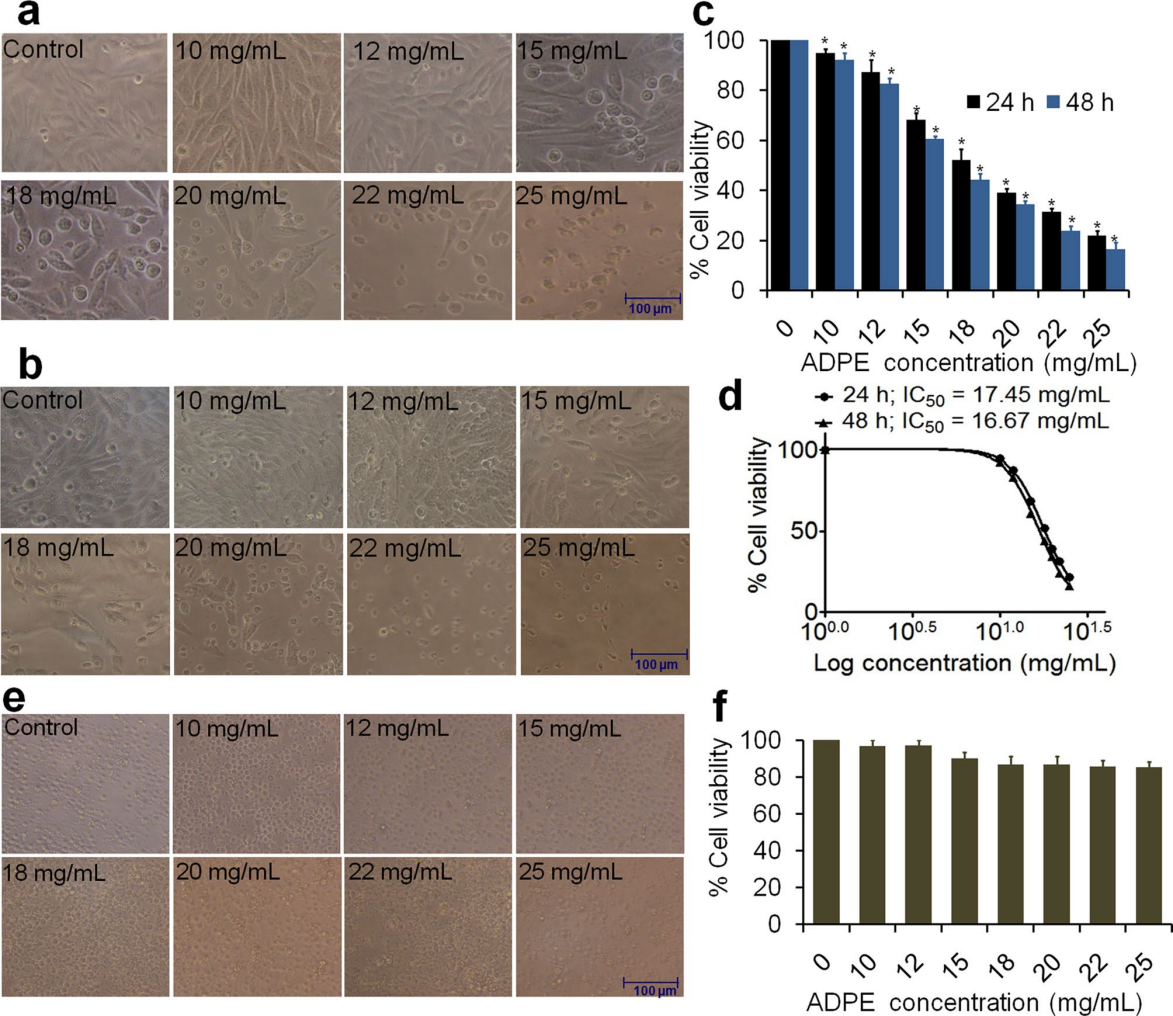
Microscopic observation and cytotoxic activity of different concentrations (10–25 mg/mL) of ADPE against MDA-MB-231 and PBMCs. (**a**) and (**b**) Photomicrograph of MDA-MB-231 cells treated with 10 to 25 mg/mL concentrations of ADPE at 24 and 48 h, respectively. Photomicrographs were taken with an inverted phase contrast microscope (Scale bar = 100 μm). (**c**) Percent cell viability of ADPE at various concentrations against MDA-MB-231 cells after 24 and 48 h incubation. (**d**) Dose response curve (Log concentration *vs* % cell viability) representing IC_50_ values of ADPE at 24 and 48 h incubation. (**e**) Photomicrograph of PBMCs treated at various concentrations of ADPE at 24 h (Scale bar = 100 μm). (**f**) Percent cell viability of ADPE at various concentrations against PBMCs after 24 h incubation. Values are expressed as mean ± SEM of three independent experiments. *p < 0.05 as compared to control.

MDA-MB-231 cells were treated with increasing concentrations of ADPE and photographed at 24 and 48 h of ADPE exposure. As shown in Fig. 2a and b, the control/untreated cells exhibited normal features such as a typical adherent and homogeneous cell surface at both 24 and 48 h of incubation. Following exposure to 10–25 mg/mL ADPE for 24 h, majority of MDA-MB-231 cells developed a non-adherent, detached and rounded morphology. Moreover, ADPE increased the typical morphological apoptotic variations in MDA-MB-231 cells at 48 h incubation; showing both dose-and time-dependent activities. As indicated in Fig. 2c, ADPE reduced cell viability to 94.8, 87.3, 68.2, 52, 39.1, 31.4 and 21.8% at 10, 12, 15, 18, 20, 22 and 25 mg/mL, respectively at 24 h incubation period. ADPE exerted a more pronounced cytotoxic effect at 48 h, reducing the viability of treated MDA-MB-231 cells to 92.1, 82.7, 60.7, 44.1, 34.3, 23.8 and 16.3%, respectively at the respective doses of ADPE. Thus, the cell viability data suggested that ADPE treatment significantly reduced MDA-MB-231 cell growth in both dose- and time-dependent manner. The IC_50_ value of ADPE was found to be 17.45 and 16.67 mg/mL after 24 and 48 h exposure, respectively (Fig. 2d). ADPE was also used to check the cytotoxic effect on normal human PBMCs. Interestingly; ADPE did not exhibit any significant toxicity against human PBMCs (Fig. 2f). The morphological features of PBMCs at different concentrations of ADPE also validated the data of cytotoxic assay, where no significant variations were found in number and morphology of normal cells (Fig. 2e). These results suggest that ADPE is not toxic to normal human cells in the range tested.

### Stimulation of chromatin condensation and induction of apoptosis by ADPE

**Figure 3:**
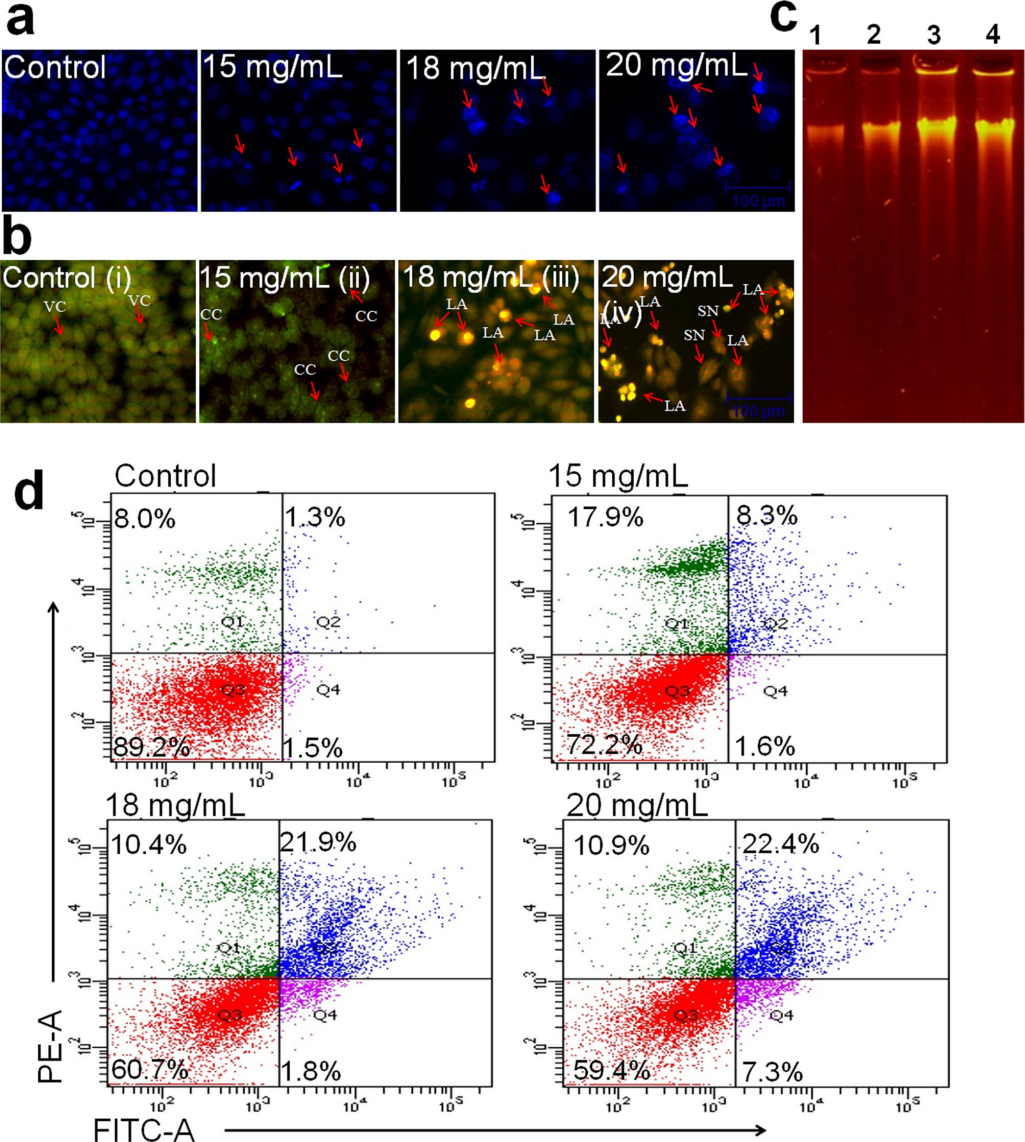
Chromatin condensation and apoptosis-inducing activity of ADPE in MDA-MB-231 treated cells (**a**) Chromatic condensation of MDA-MB-231 treated cells at 15, 18 and 20 mg/mL of ADPE for 48 h by Hoechst 33342 staining (Scale bar = 100 μm). (**b**) Fluorescent micrographs of AO/PI-double-stained MDA-MB-231 cells at 15, 18 and 20 mg/mL of ADPE at  48 h. (i) Untreated MDA-MB-231 cells depict normal  structure (ii) Early apoptosis features such as chromatin condensation and membrane blebbing were observed at 15 mg/mL of ADPE (iii) Late apoptosis cells were observed at 18 mg/mL (iv) Late apoptosis and secondary necrosis were observed at 20 mg/mL of extract (Scale bar = 100 μm), VC: Viable cells; CC: Chromatin condensation; LA: Late apoptosis and SN: Secondary necrosis . (**c**) DNA fragmentation assay in MDA-MB-231 cells as an index of apoptosis. Lane 1: showing control MDA-MB-231 cells; Lane 2, 3, and 4: cells treated with 15, 18, and 20 mg/mL of ADPE, respectively. (**d**) Flow cytometry analysis of MDA-MB-231 cells after 48 h of treatment using annexin V/FITC & PI double stain. Representative figures showing the population of viable (annexin V^−^ PI^−^), early apoptotic (annexin V^+^ PI^−^), late apoptotic (annexin V^+^ PI^+^) and necrotic (annexin V^−^ PI^+^) cells.

As is evident from the photomicrograph (Fig. 3a), ADPE increased the chromatin condensation in MDA-MB-231 cells stained with Hoechst 33342 depending upon dose. Doses 15 and 18 mg/mL showed less condensation of nucleus whereas 20 mg/mL of ADPE exhibited maximum nuclear condensation. As shown in AO/EtBr staining, control cells appeared live and healthy having uniformly green-colored nucleus, while treated cells appeared to be either in early apoptosis (green-colored with condensed nuclei) or in late apoptosis stage (orange-red colored cells with condensed nuclei). Higher doses of ADPE increased the late apoptotic features (Fig. 3b). ADPE was further analyzed to determine DNA fragmentation in MDA-MB-231 cells. Intact undamaged DNA band was obtained in control well, while treated cells displayed progressive DNA damage and fragmentation depending upon dose (Fig. 3c). ADPE at 15 mg/mL showed less shearing of DNA but it was found to be increased at 20 mg/mL of extract.

### Quantification of early and late apoptosis induced by ADPE

To quantify early and late apoptosis, MDA-MB-231 cells were evaluated further by Annexin V-FITC Apoptosis Detection Kit (Biovision, USA). Untreated cells showed 89.2% live and an intact physical shape whereas ADPE at 15 mg/mL increased cell death by decreasing live cells percentage to 72.2% and enhanced percentage of early apoptotic (1.6%) and late apoptotic (8.3%) cells (Fig. 3d). The doses 18 and 20 mg/mL caused significant increase in percentage of late apoptotic cells to 21.9 and 22.4%, respectively. A small proportion of dead cells (8.0%) were detected in controls, which may be attributed to floating of detached cells after MDA-MB-231 overgrowth post 48 h.

### Intracellular ROS generation by ADPE

**Figure 4:**
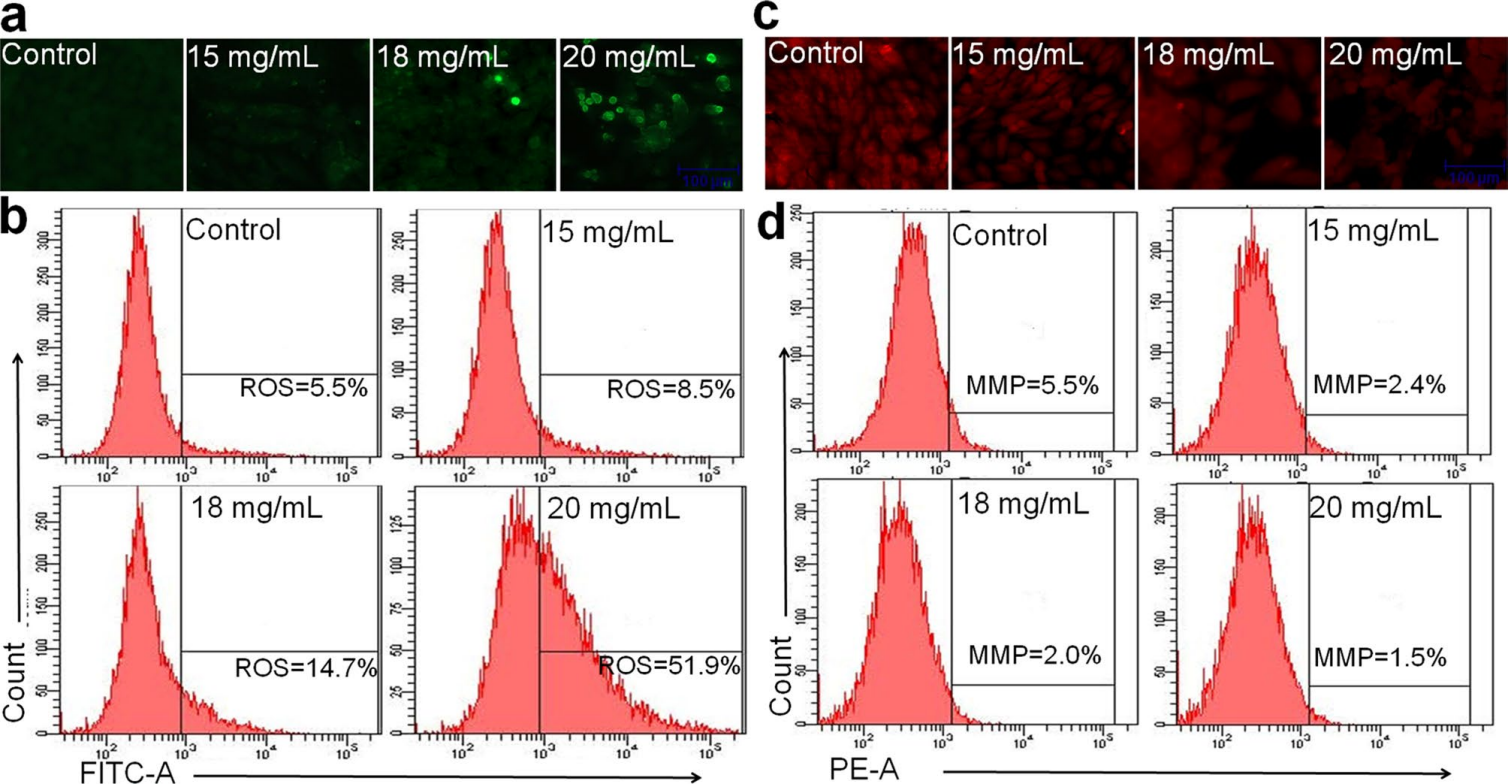
Intracellular ROS generation and mitochondrial membrane potential of human MDA-MB-231 cells (**a**) Photomicrographs showing intracellular ROS generation induced by three effective concentrations (15, 18 and 20 mg/mL) of ADPE after 12 h incubation. Photomicrographs were taken with a fluorescence microscope (Scale bar = 100 μm). (**b**) The fluorescence in the cells is represented as the percentage of ROS production analyzed using flow cytometry. (**c**) Photographs indicate a decrease in MMP, an early event in apoptosis with increased concentrations of ADPE. Photomicrographs were taken with a fluorescence microscope (Scale bar = 100 μm). (**d**) Fluorescence in the cells is represented as the percentage of MMP reduction in MDA-MB-231 cells analyzed by flow cytometry.

Flow cytometry analysis of ROS measurement revealed that the level of ROS intensity in control MDA-MB-231 cells was 5.5%. This small amount of ROS level shows the characteristic feature of live  cells. ROS levels were enhanced by 8.5 and 14.7% at 15 and 18 mg/mL of ADPE as compared to control. Moreover, ROS production in treated cells was increased enormously by 51.9% at 20 mg/mL of ADPE (Fig. 4b). The fluorescent microscopy analysis of ROS intensity was found to be consistent with flow cytometry data (Fig. 4a).

### Decrease of MMP by ADPE

As is evident from photomicrograph (Fig. 4c), the red fluorescent intensity of dye Rh123 was inversely proportional to increasing doses of ADPE due to loss of MMP in treated cells. Flow cytometry analysis depicted the percent level of MMP at various doses of ADPE (Fig. 4d). The untreated MDA-MB-231 cells exhibited MMP of 5.5% whereas it was decreased to 2.4, 2 and 1.5% at 15, 18 and 20 mg/mL of ADPE, respectively. Results suggest that ADPE causes a dose-dependent decrease in MMP in treated MDA-MB-231 cells.

### Induction of cell cycle arrest at S and G_2_/M phase by ADPE

**Figure 5:**
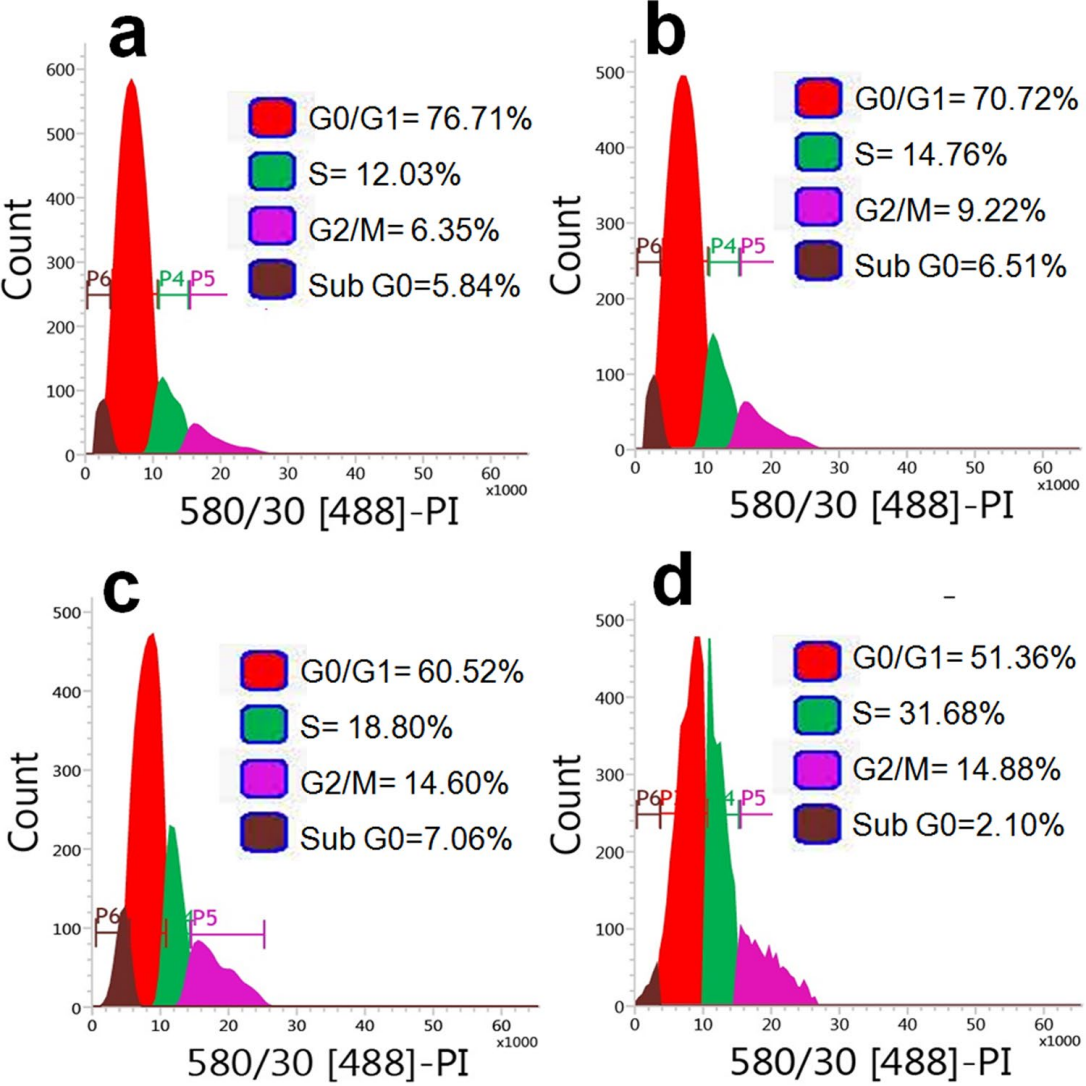
DNA content analysis by flow cytometry. Pictorial graph showing the mean proportion of cells in different phases of cell cycle (**a**) Untreated control (**b**) 15 mg/mL (**c**) 18 mg/mL and (**d**) 20 mg/mL of ADPE at 48 h.

MDA-MB-231 cells were treated with ADPE for 24 h and then subjected to cell cycle analysis using flow cytometry. As revealed by cell cycle analysis, ADPE substantially increased the number of MDA-MB-231 cells in the S phase which was accompanied by a proportional decrease in the percentage of cells in the G0/G1 phase (Fig. 5a–d). ADPE also partially increased the proportion of cells in the G2/M phase of the cell cycle. These results indicate that ADPE arrested the cell cycle of MDA-MB-231 cells in both S and G2/M phase.

### Downregulation of AKT/mTOR signaling and  downstream modulation of Bcl-2 family proteins by ADPE

**Figure 6:**
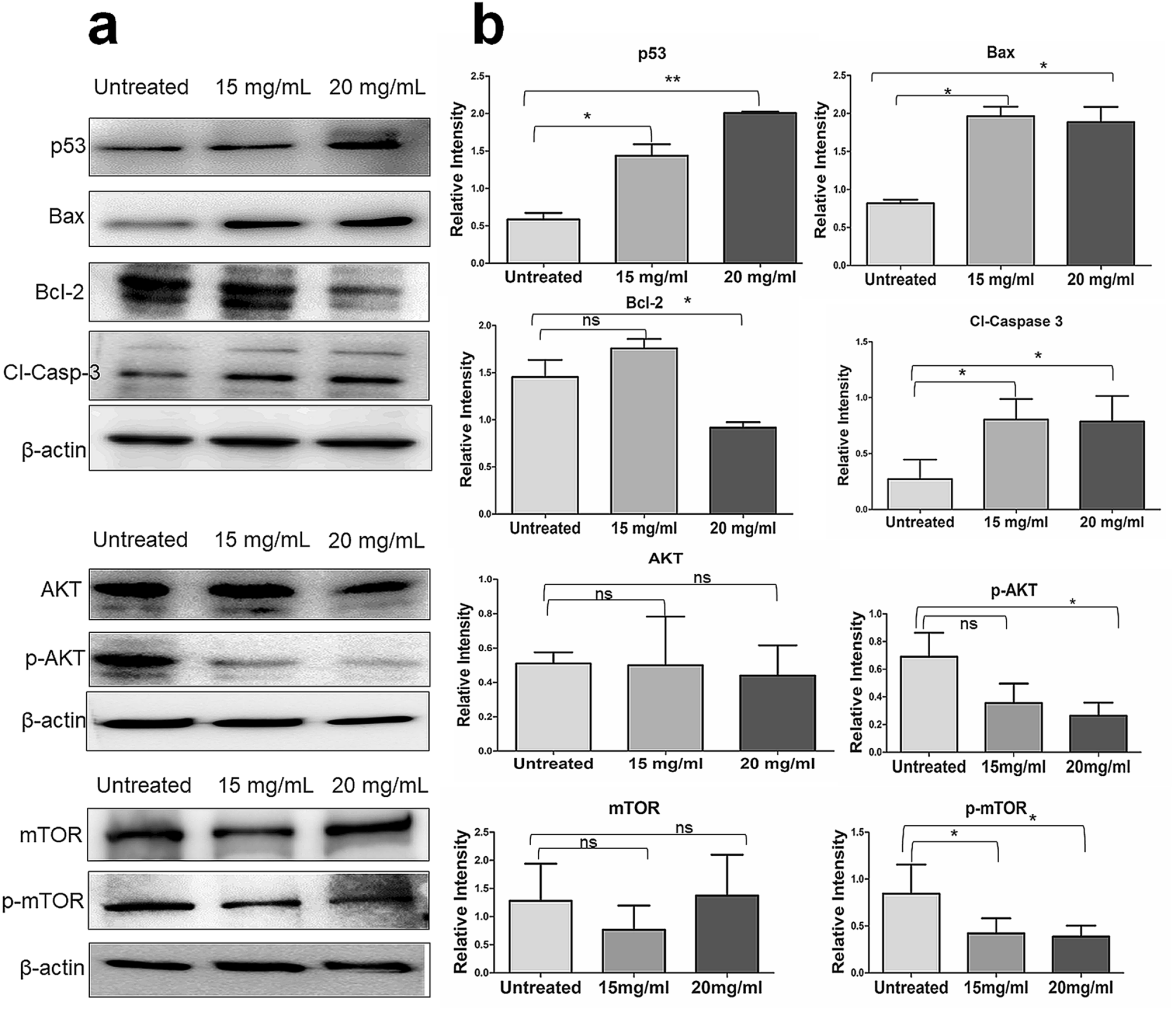
Western blot analysis of apoptotic proteins and signaling molecules in MDA-MB-231 cells. (**a**) Immunoblot analysis showing expression levels of p53, Bax, Bcl2, cleaved Caspase-3, mTOR, p-mTOR, AKT and p-AKT. The MDA-MB-231 cells were treated at two effective concentrations of ADPE (15 and 20 mg/mL) for 48 h. Equal amounts of total proteins (30 μg/lane) were subjected to 10–12% SDS-PAGE. Expression of p53, Bax, Bcl2, cleaved Caspase-3, mTOR, p-mTOR, AKT, p-AKT and β-actin were detected using specific antibodies. Lane 1: 0 mg/mL (Untreated); Lane 2: 15 mg/mL; Lane 3: 20 mg/mL of ADPE. (**b**) Graph representing relative intensity of various proteins *vs* concentration. The data represents mean ± SEM of three independent experiments.

To investigate the mechanisms underlying ADPE-induced cell death, immunoblot analysis was carried out for the expression of key markers such as tumor suppressor p53, pro-apoptotic Bax, anti-apoptotic Bcl-2, and effector caspase i.e. cleaved caspase-3. Further, the expression of mTOR and AKT molecules were investigated to analyze the intracellular signaling pathway. Results showed that Bax was upregulated and Bcl-2 was down-regulated in ADPE treated cells. The expression of p53 and cleaved caspase-3 was increased in MDA-MB-231 cell line following 48 h of ADPE treatment. Moreover, the results demonstrated that both p-AKT and p-mTOR levels were reduced in MDA-MB-231 cells following 48 h of ADPE treatment (Fig. 6). The raw data of western  blots showing various signaling molecules in the apoptosis of MDA-MB-231 cells are  shown in Figure S1. These results indicate that ADPE inhibits AKT/mTOR signaling pathway important in regulating cell growth and proliferation of  breast cancer cells.

## Discussion

Amongt breast cancer, TNBC is the most dangerous  with increased risk of cancer progression and poorer prognosis due to lack of targeted hormonal therapy.  MDA-MB-231 is a TNBC cell line as it does not express estrogen, progesterone and HER2 receptors. Herbal extracts and their isolated molecules have been found to be effective against various cancers^21^. In the present study, the evaluation of mitochondrial-mediated apoptosis, cell cycle arrest and underlying intracellular signaling pathways of cell death by ADPE against human TNBC MDA-MB-231 cells has been reported for the first time.

The anti-proliferative effect of ADPE on MDA-MB-231 cell line has been shown in Fig. 1. The MTT results revealed  that exposure of MDA-MB-231 cells to ADPE resulted in growth inhibition of cells in a dose- and time-dependent manner. ADPE showed IC_50_ values of 17.45 and 16.67 mg/mL at 24 and 48 h, respectively. ADPE was also tested on normal human PBMCs to ascertain its toxicity. Interestingly, ADPE was found to be non-toxic without any associated morphological effects on PBMCs (Fig. 2e and f), which suggests that ADPE is non-toxic to normal human cells. As majority of the phytocomponents present in the pulp of Ajwa dates were characterized as polysaccharides in LC–MS, therefore, larger proportions are needed to elicit biological efficacy of ADPE. This study suggests that consumption of large portions of Ajwa dates may be conducive to lead a healthy and cancer-free life. The morphological data under inverted phase-contrast microscopy revealed the natural and fibroblastic morphology of untreated TNBC cells. ADPE caused a decrease in the number of cells by an alteration in their shape and adherence. These events represent the classical features of early apoptosis^22^. Further, to confirm apoptotic cell death, MDA-MB-231 cells were investigated using Hoechst 33342 nuclear stain under a fluorescence microscope. The treated cells displayed cell shrinkage, blebbing of plasma membrane without loss of integrity, nuclear condensation and formation of pyknotic bodies (Fig. 3). Further, AO/EtBr double stain was used to analyze the early and late apoptosis in ADPE treated cells. In early apoptosis, AO binds within the fragmented DNA of the cells emanating bright green fluorescence, while in late apoptosis; PI binds to denatured DNA displaying reddish-orange color. This study suggests that lower doses of ADPE lead to early apoptosis while higher doses lead to the late stages of apoptosis. The preliminary assays such as nuclear condensation assay, AO/EtBr assay and DNA fragmentation assay are the initial confirmation of apoptotic/necrotic cell death. Many previous studies have tested these preliminary assays to validate the induction of cell death via apoptosis or necrosis^23–25^. Therefore, based on these preliminary tests, Annexin V-FITC& PI double staining assay was carried out to quantify the percent of early and late apoptosis and necrotic cells. Flow cytometry analysis of Annexin- V/FITC & PI  double stain depicted that the percentage of viable cells decreased with a concomitant increase in the percentage of cells undergoing early and late apoptosis. At a low dose, ADPE treatment resulted in early apoptotic cells while late apoptotic stages were found at higher doses (Fig. 3). This data suggests that ADPE treatment pushes the cancer cells into late apoptosis stage. A previous study has also reported that methanolic extract of Ajwa dates induces apoptosis in breast cancer MCF-7 cells by increasing the percentage of cells in late apoptosis^26^. DNA fragmentation data also confirmed the apoptosis-inducing efficacy of ADPE against MDA-MB-231 cells.

The regulation of intracellular ROS levels is crucial in maintaining cellular homeostasis and thus, different ROS levels can cause diverse biological responses. At low levels, ROS act as signaling molecules while at high levels they induce cell damage and death^27^. Therefore, the generation of ROS levels in treated cells was examined using DCFH-DA stain through fluorescence microscopy and flow cytometry. Results revealed that ADPE increased  the level of ROS generation in a dose-dependent manner (Fig. 3). Excess cellular levels of ROS are responsible for damaging the various biomolecules such as proteins, lipids, nucleic acids, cell membranes and organelles which may result in progressive cell dysfunctions and cellular apoptosis^27^. Oxidative stress can disrupt the balance between ROS production and radical-scavenging leading to loss of MMP and release of cytochrome c from the inner mitochondrial membrane  resulting in cellular apoptosis^28^. Thus, ADPE has been found to decrease MMP in MDA-MB-231 cells in a dose-dependent manner (Fig. 4). Excessive ROS production can activate various signaling molecules in cancer cells which initiate cell cycle arrest and apoptosis^29^. The cell cycle data revealed that ADPE treatment of MDA-MB-231 cells resulted in arrest of cells in S phase and sparingly in the G2/M phase with an accompanying decrease in the G0/G1 phase (Fig. 5). This study confirmed that ADPE interferes the initiation of DNA replication and thus arrests MDA-MB-231 cells at the S phase, while cells arrested in G2/M phase are restricted by ADPE to repair damaged DNA before entering mitosis. A previous study has also shown that *Allium atroviolaceum* flower extract induces S and G2/M phase arrest in MCF-7 and S phase arrest in MDA-MB-231 cells^30^.

ROS cause cell cycle arrest and induce apoptosis by activating various signaling cascades and signal-regulating kinase pathways^27,31^. Apoptosis also requires permeabilization of the outer mitochondrial membrane which is controlled by the Bcl-2 family proteins. Therefore, the present study also attempted validation of our hypothesis on the modulation of the Bcl-2 family proteins and signal-regulating AKT/mTOR pathway in the ADPE-extract mediated apoptosis against MDA-MB-231 cells. For this, protein expression of p53, Bax, Bcl-2 and cleaved caspase-3 was analyzed along with the expression of p-AKT and p-mTOR in ADPE- treated MDA-MB-231 cells. Protein expression data revealed that ADPE increased the expression of tumor suppressor p53, pro-apoptotic Bax, and effector cleaved caspase-3 and reduced the expression of anti-apoptotic Bcl-2 protein (Fig. 6). Tumor suppressor p53 gene, also called guardian of the genome, plays an important role in cell growth inhibition and induction of apoptosis after DNA damage. After DNA damage, p53 gene is activated and it promotes high expression of pro-apoptotic regulator Bax and low expression of anti-apoptotic gene Bcl-2. Eventually, the modulation of the Bcl-2 family proteins initiates cascade reaction of caspases leading to caspase-3 activation and resulting in nuclear apoptosis^22,27^. Thus, our results clearly show that ADPE exhibited apoptosis via intrinsic pathway.

Growth factors can suppress apoptosis and regulate cell growth and cell survival in a transcription independent manner by activating the serine/threonine specific protein kinase, AKT^32^. Activated AKT, also known as Protein kinase B (PKB), translocates to the cytoplasm and nucleus and activates a number of downstream targets following activation of mTOR^33^. The mammalian target of rapamycin (mTOR) functions as a serine/threonine protein kinase, which can affect gene transcription and translation by regulating cell growth, cell proliferation and cell survival^34^. AKT promotes cell survival by inhibiting apoptosis, and thus it can be said that AKT is downregulated during apoptosis processes. Therefore, due to suppression of the AKT/mTOR pathway, cells lose their survival and proliferation capability; which may trigger programmed cell death, including apoptosis, autophagy and necroptosis. Interestingly, our protein expression data revealed that the expression level of p-AKT and p-mTOR underwent a down regulation after 48 h of ADPE treatment in  MDA-MB-231 cells (Fig. 6). A previous study has also reported that *Murraya koenigii* leaves extract induced mitochondrial apoptosis in DLD-1 colon cancer cells by downregulating mTOR/AKT pathway^35^. LC–MS data revealed the presence of carbohydrates containing disaccharides and their derivative along with phenolics, flavonoids, phytosterols and carotenoids in ADPE (Table 1). The identified bioactive components such as maltose, catechin, myricetin, quercetin, β-sitosterol, digalacturonic acid, chlorogenic acid and β-carotene are known to exhibit various pharmacological activities such as antioxidant, anti-inflammatory, antimicrobial, hepatoprotective and anticancer^36,37^.

## Conclusions

**Figure 7:**
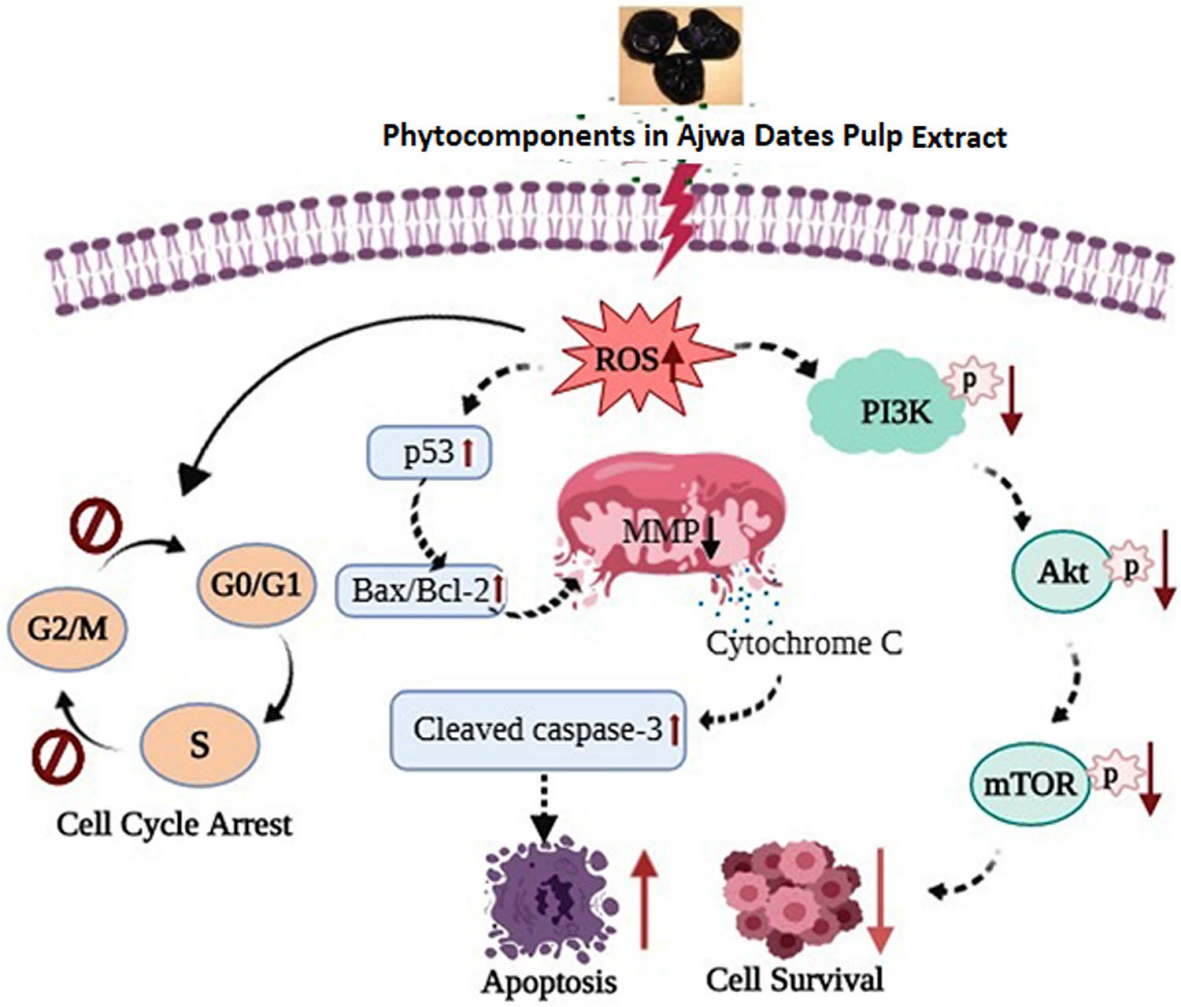
Schematic diagram summarizing the proposed mechanism of ADPE action on MDA-MB-231 cells. Bioactive components from ADPE arrest G2/M and S phase checkpoints and induce apoptosis by upregulation of p53, Bax and cleaved caspase-3, thereby leading to downregulation of Bcl-2 family proteins and AKT/mTOR pathway.

In conclusion, the present study revealed the potent growth-inhibitory effect of Ajwa dates pulp against human TNBC MDA-MB-231 cells with no significant toxic effect on normal human PBMCs. The growth-inhibitory effect was found to be associated with ROS generation, MMP depletion, cell cycle arrest, upregulation of tumor suppressor p53, pro-apoptotic Bax, and effector cleaved caspase-3 and downregulation of anti-apoptotic Bcl-2 protein, p-AKT and p-mTOR signaling molecules. Figure 7 summarizes the proposed mechanism of action underlying the anticancer effect of ADPE against MDA-MB-231 cells. The current research has focused on a single TNBC cell line and not all TNBC cell lines or triple-negative breast cancer cases. Furthermore, the cytotoxicity test was carried out on normal human PBMCs rather than a normal mammary cell line. As a result, further research into the mechanism of ADPE is needed on all TNBC cell lines and normal mammary cells. Based on the findings of this study, ADPE has the potential to be developed into a novel and effective anticancer drug against human breast cancer in future, and/or as a potent adjunct to the mainline of breast cancer therapy. However, further clinical trials are required to confirm the therapeutic basis of drug development.

## Supplementary Information

Supplementary Information.
