# Supplementary material for: Phytochemicals from Ajwa dates pulp extract induce apoptosis in human triple-negative breast cancer by inhibiting AKT/mTOR pathway and modulating Bcl-2 family proteins
Source: Sci Rep. 2021 May 14;11:10322. doi: 10.1038/s41598-021-89420-z (PMC8121835; doi:10.1038/s41598-021-89420-z)

**Supplementary Figure**

**Figure S1.** The raw data of protein blots showing various protein markers in the apoptosis of MDA-MB-231 cells.


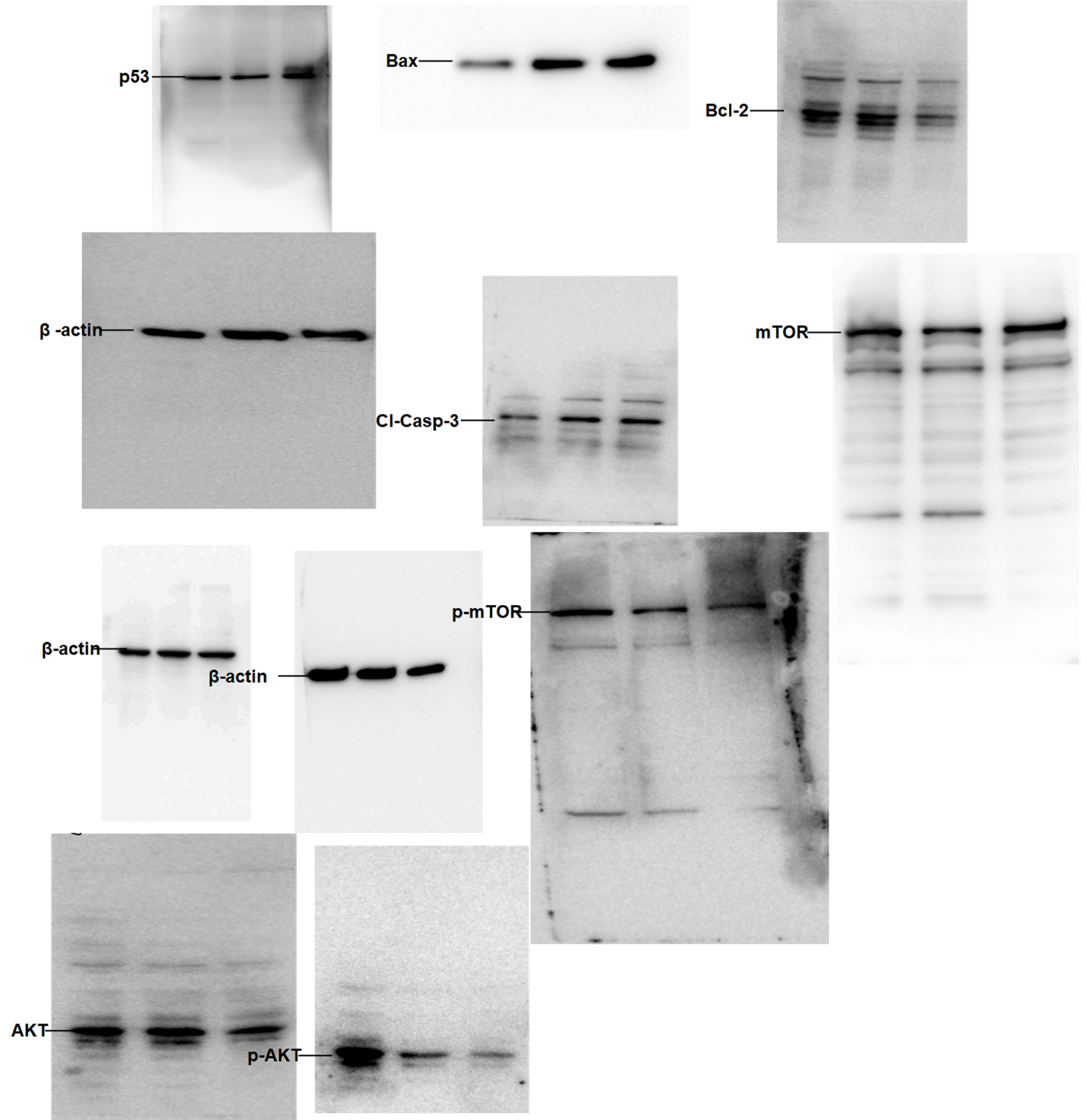

Supplement: Supplementary file 1 — Supplementary Information. [file 41598_2021_89420_MOESM1_ESM.docx]
